# Training Proposal in Palliative Care for Primary Care Nurses in a Health Area in Spain

**DOI:** 10.3390/nursrep13020078

**Published:** 2023-06-11

**Authors:** Isidro García-Salvador, Encarna Chisbert-Alapont, Amparo Antonaya Campos, Jorge Casaña Mohedo, Clara Hurtado Navarro, Silvia Fernández Peris, José Bonías López, Maria Luisa De la Rica Escuín

**Affiliations:** 1 Nurse Oncology Service, Valencia Health Department, Doctor Peset, 46017 Valencia, Spain; isidro.gs@hotmail.com; 2 Foundation for the Promotion of Health and Biomedical Research in the Valencian Region (FISABIO), 46020 Valencia, Spain; camposampa@gmail.com (A.A.C.); jcasamo@gmail.com (J.C.M.); klaracha50@gmail.com (C.H.N.); silviafp9@gmail.com (S.F.P.); boniasjose@gmail.com (J.B.L.);; 3 Research Group INCUE, Valencia Health Department, Doctor Peset, 46017 Valencia, Spain; 4 Nurse Hematology Service, Valencia Health Department La Fe, 46026 Valencia, Spain; 5 Primary Care Nursing Director, Valencia Health Department, Doctor Peset, 46017 Valencia, Spain; 6 Nursing Department, Faculty of Health Sciences of Universidad Internacional de Valencia and Faculty of Medicine and Health Sciences of Universidad Católica San Vicente Mártir, 46001 Valencia, Spain; 7 Nurse Training Service, Valencia Health Department, Doctor Peset, 46017 Valencia, Spain; 8 Carena Association of Psycho-Oncology, Valencia Health Department, Doctor Peset, 46017 Valencia, Spain; 9 Nurse Primary Care Center of San Marcelino, Valencia Health Department, Doctor Peset, 46017 Valencia, Spain; 10 Nurse Research Group on Care in End-of-Life Processes, Institute for Health Research Aragón, 50009 Zaragoza, Spain

**Keywords:** primary care, nursing, palliative care, nurse training, bereavement care

## Abstract

Background: Primary Health Care nurses express deficits in their training in Palliative Care. The purpose of this study is to design a Palliative Care training plan and a bereavement care protocol for Primary Health Care nurses of the Dr. Peset Health Department according to their needs. Methods: Assessment of theoretical and practical training needs and literature review for the design of the training plan. Results: A training plan was elaborated that included a protocol of care for the bereaved. The plan was adjusted to the needs detected in Primary Health Care nurses of the Dr. Peset Health Department. Important training deficits were detected in clinical practice; Conclusions: Improving the care of people with palliative needs in Primary Health Care requires adequate training of the nurses who care for them so their knowledge is the basis of their interventions. This study was not registered.

## 1. Introduction

The Spanish national strategy for Palliative Care (PC) [1] states that people with palliative needs should be attended to by Primary Health Care (PHC) teams. The intervention of specific PC teams will only be necessary in complex situations [2].

The Health Department of Valencia-Dr. Peset serves a population of 284,774 inhabitants [3]. In order to facilitate the coordination of health care, this department is divided into 11 geographic health areas, which include 11 health centers and 10 auxiliary clinics [4], where 173 primary care nurses work.

PHC professionals are in an ideal position to identify the need for PC and initiate it early [5], and may be predisposed to provide it [6,7]. Moreover, given their proximity, these nurses establish a bond with individuals that favors continuity of care. However, studies indicate that PHC nurses do not feel prepared, or express deficits in their PC training [8,9,10]. These basic training deficits, especially at the practical level, are also expressed by other nurses at hospital level [11,12]. This may be due to the unequal training that Spanish PC nurses receive during their undergraduate studies [13]. This problem also seems to be present in other countries and different organizations, nationally and internationally, that have designed curricula for nurse training in PC. The End-of-Life Nursing Education Consortium (ELNEC) offers undergraduate and postgraduate courses aimed at improving palliative nursing care and is supported by the American Association of Colleges of Nursing [14]. Other programs are interdisciplinary and are aimed at PHC professionals, such as the Gold Standards Framework in the United Kingdom [15] or the CAPACITI Training Program in Canada [16]. In Spain, the Spanish Association of Palliative Care Nursing (Spanish acronym AECPAL) made recommendations on the PC training that nurses should receive in their undergraduate studies, considering it as part of basic training, essential to provide nurses with the necessary competencies to care for people with palliative needs [17]. This recommendation is based on the PC nursing competencies previously published by the Spanish Society of Palliative Care (Spanish acronym SECPAL) [18].

The theoretical competencies of palliative care nurses are horizontal for all nurses, regardless of their field of practice [18]. However, their practical application may vary. In the competency of managing and coping with the bereavement process, PHC or specific home palliative care resources nurses can perform bereavement follow-up; however, hospital nurses can only perform complicated bereavement prevention. It is estimated that 10–30% of bereaved individuals will experience complicated bereavement [19,20,21] and require specialized care. Furthermore, without adequate prevention and follow-up, 7% of complicated bereavement will eventually develop into pathological bereavement [22]. Here lies the importance of follow-up of the bereaved by PHC nurses, to identify risk factors for complicated bereavement [23] and the manifestations of normal bereavement [24,25,26].

Moreover, the existence of protocols allows the identification of palliative care needs and standardization of professional care. The absence of protocols or lack of knowledge thereof can be one of the reasons for neglect or deficient care in some of the nursing competencies, such as care of the bereaved [10]. Including them in the training plan can encourage care and help nurses in their follow-up of family members after a patient’s death, by establishing visiting times and the tasks for each visit.

Additionally, it has been shown that some nurses possess theoretical knowledge in PC, but do not apply it in their clinical practice [10,27]. This justifies the recommendation for specific training for groups and specific health areas, aimed at the development of nursing competencies that will modify attitudes and favor the applicability of knowledge [12,28]. Furthermore, although the current trend is towards online training, some authors defend the need for face-to-face training, learning with simulation or case resolution, and discussion groups, which support the transfer of knowledge to behavior in care [11,27,27,29,30].

A training plan tailored to the actual needs of PHC nurses requires a prior assessment of such needs. Both theoretical knowledge and its practical application should be evaluated. Some instruments such as the PCQN [31] and Rotterdam MOVE2PC [32] do not assess practical applicability. Others assess self-competence without assessing whether there is transfer to practice [33], or assessing competencies in various disciplines [30]. The INCUE instrument was specifically designed to measure theoretical and practical training needs among PHC or home care nurses [34]. It is necessary to not only acquire knowledge, but also to apply it so that it reaches the population. Therefore, targeting specific training based on the gaps detected by the INCUE instrument in these nurses could have an impact on the provision of more effective PC for patients and their families [34].

The general objective of the following study was to develop a training plan in PC for PHC nurses of the Valencia-Dr. Peset Health Department. The specific objectives were to identify the training needs of these nurses and to develop a bereavement care protocol as part of the content of the training plan.

## 2. Materials and Methods

### 2.1. Design

The general objective was approached in several steps, complementary to each other, and which are presented sequentially to facilitate their understanding.

In the first step, a systematic review of the documents published on basic training in palliative care for nurses was carried out by a group of 10 experts, until consensus on the design of the general lines of the training program was reached.

In the second step, a cross-sectional descriptive study was designed to assess training needs of primary care nurses in the Valencia-Dr. Peset Health Department (Spain).

The third step involved the protocol development, considering the needs identified in the second step and a review of existing nursing protocols in the Department and the existing care and referral routes for patients to different levels of care. Bereavement care protocols from other Spanish health departments and home care settings were also reviewed. 

### 2.2. Sample and Data Collection

The first step involved the following. To guide the design and general lines of content in the training program, a bibliographic search was carried out using Scopus, Pubmed, Lilacs, Cuiden and Cinahl databases. The search criteria were narrowed to publications within the last 10 years (2012–2022) and to the descriptor terms “palliative care”, “training” and “nursing”. Documents from the gray literature related to the above descriptors were also included. 

Experts involved in the design of the training program were selected on the basis of their knowledge and experience in palliative care. Each of them possess advanced training in PC and teach PC in a clinical setting, with a minimum of 5 years experience.

The group of external reviewers each possess advanced training in PC and at least 10 years of practice experience in the primary care setting. 

The second step is detailed as follows. For the training needs assessment, studying the entire population of primary care clinical nurses in the department was initially considered; however, despite repeated reminders to participate, this was not possible. A representative sample was chosen, and the sample size requirement was set at 92 participants out of a total of 173 nurses, assuming 95% confidence, 3% precision, *p*-ratio = 0.5 (5%) and expected losses of 15%. Pediatric nurses without adult care responsibilities and midwives were exclusion criteria. A total of 102 responses were included. 

Sampling was non-probabilistic, snowball sampling. Recruitment was carried out through the coordinators of the department’s health centers, with reminders about participation every 15 days, for a total of 9 reminders. Possible coercion during recruitment was prevented by blinding coordinators to the number of responses from the nurses under their charge, and with reminders of a general scope.

Data were collected via an anonymous online questionnaire on Google Forms, from January to May 2022. The self-report questionnaire contained a brief introduction, including the objective of the study, inclusion criteria and the need for consent for participation, guaranteed anonymity, confidentiality and the possibility of withdrawal.

The widespread access to the Internet and the professional use of the same IP allowed all strata of the study population to be represented, avoided self-selection bias and ensured the safety of all participants. Furthermore, the participants presented similar proportions to the total population in relation to sex, mean age and education, according to the data provided by the department’s statistics service, which allows us to consider the results as representative of the population.

Third step’s methods were as follows. Existing bereavement care protocols and care routes were reviewed by the research group. The protocols were obtained from the different Spanish health services or their web pages. The care routes were provided by the Valencia-Dr. Peset Health Department. A systematic review was also performed following the same method as for the design of the training plan, but using the descriptors “grief”, “bereavement”, “protocol” and “nursing” in different combinations. The protocol was developed by the research group and coordinated by the principal investigator and the group psychologist, integrating the care pathway. It was subsequently reviewed by three psychologists, four nurses and one physician, all experts in PC and bereavement. The final version of the protocol was also submitted to the Department’s PC Clinical Committee for approval.

### 2.3. Instruments

The aim of training is to provide nurses with the knowledge, skills, attitudes and judgment associated with their profession and field of expertise, that are necessary to adequately solve the problems that their professional practice involves [35].

The development of a training program can be approached from different perspectives [36,37,38], with common elements in them. In this study, the training program was designed according to the following stages based on the different approaches:

1. Analyze of the situation at the time of cutoff.
2. Establish the professional profile at which the plan is aimed.
3. Establish the objectives of the plan.
4. Decide on the educational strategy and teaching methodology.
5. Design the syllabus and content of the training plan.
6. Define the profile of the teachers and select them according to the resources available.
7. Establish the evaluation systems of the results.
8. Determine the timing of the training activity.
9. Prepare a report on the estimated cost of the plan.
10. Draft the training plan.

The first stage of the design of the training program was carried out by assessing training needs using the INCUE instrument in the period described [34]. This instrument assesses knowledge of PC and its application in clinical practice in five areas. This is unlike other, more widely used instruments, such as the PCQN [31], in which the questions focus on symptomatic control and only on theoretical competencies without assessing their practical application. Others, such as the MOVE2PC [32] and the Palliative Care Nursing Self-Competence (PCNSC) scale [33], assess the subjective perception of competence in certain situations, without actually assessing whether it is achieved. In contrast, the INCUE instrument was created exclusively to measure theoretical and practical training needs in primary care nurses, and specifically assesses the following areas: PC principles, symptom management and care plans, coping with loss and death, communication skills, and ethical and legal aspects. Knowledge is assessed by answers to dichotomous Yes/No questions and practical application is measured by answers to a 5-point Likert scale (Never-Always). The minimum score to assess sufficient theoretical training is set at 18 correct answers out of 23 questions. The minimum total score to assess practical training is set at 90 points out of a possible 120; scoring 0 for “never”, 1 for “rarely”, 2 for “sometimes”, 3 for “frequently” and 4 for “always” [34].

Other variables analyzed were training in palliative care and the perceived need for preparation and training in palliative care (by multiple response). Also included in the questionnaire was a question on knowledge of the PC care protocols in their work center.

The professional profile matched the inclusion criteria of the nurses recruited. 

Stages 3 to 7 of the design of the training program were developed by the group of experts through two rounds of consensus, based on the needs detected through the INCUE instrument, the review of the documents found in the literature search and the AECPAL recommendations on undergraduate training in palliative care [17] that were considered as basic training. Subsequently, the document was subjected to an external review using the Delphi method.

The schedule of the training sessions was established by the research group in coordination with the Department’s Nursing Management during working hours and with the least possible impact on citizens’ service.

The cost estimate for the implementation of the plan was calculated by the research team based on the training plan prepared by the experts and the existing resources in the Department. 

The training plan was drafted by the principal investigator.

### 2.4. Data Analysis

A descriptive analysis was performed according to the grouping variables for the quantitative data, obtained from the training needs assessment questionnaire. Mean and standard deviation were calculated for quantitative variables, and frequency and proportions for qualitative variables.

Bivariate contrast tests were also performed to accompany the descriptive analysis. The Wilcoxon–Mann–Whitney test or the Kruskal–Wallis test was used, depending on whether the comparison was between two or more groups, to compare quantitative variables, and the χ2 test with *p*-value simulation (using 2000 simulations) was used to compare qualitative variables.

All analyses were performed using the R statistical environment, version 4.2.2 [39].

The documents and protocols from the literature search were screened for quality using the Spanish Critical Appraisal Skills Programme (CASPe) [40].

### 2.5. Ethical Considerations

The present study was reviewed and approved by the Drug Research Ethics Committee of the University Hospital Dr. Peset (Research Project EAPCP19-V01 and CEIM code 11/20). Potential study participants were provided with a detailed description of the study and were assured of confidentiality. Written informed consent was obtained from each participant. They were also informed of the voluntary nature of the study participation and completion without any negative consequences.

## 3. Results

### 3.1. Training Needs

Most of the participants were women, mostly graduates (71%) with an average of 21 years of professional experience (Table S1). 

Of the nurses surveyed, 26.47% had no training in PC and 52.89% received basic training (Table S1).

Only 23.53% felt fairly or very prepared to care for palliative patients and a similar percentage knew the care protocols of their center. 

They perceived a considerable or great need for training in 75.49% of the cases. In multiple responses, the areas of greatest demand were training in symptomatic control (69.61%), followed by psychoemotional training and training in bereavement and coping with loss (65.68%). Repondents also needed training in communication skills (51.96%), ethical aspects (41.17%), socio-familial (40.19%), PC principles (39.21%) and spirituality (24.51%).

The results of the INCUE questionnaire on training needs showed that 77.45% of the participants passed the theoretical part on palliative care knowledge, while 14.7% passed the practical part or applicability of the knowledge. It should be noted that 21.56% failed both parts. Comparing the percentages of participants who passed the knowledge part and the practical application of knowledge in the different areas, statistically significant differences were observed in all areas, with a *p*-value < 0.001 (symmetry test). 

The queries in the questionnaire refer to basic aspects of PC, and those with higher the level of training had higher scores, both theoretical and practical (Figure S1). The correlation between the scores of both parts and the level of education of the respondents was positive and statistically significant (Spearman correlation coefficient: ρ = 0.49, *p*-value < 0.001). Although there were differences are observed in all areas, the area with the greatest difference between the number of respondents who met the minimal score for knowledge and the number of those who applied the knowledge, at all levels of training, was the area of coping with loss and death (Table S2).

The highest scores on the questionnaire at both the theoretical and practical levels are obtained by subjects with postgraduate PC training or non-university training (Figures S2 and S3).

There was a significant contrast in the scores of the theoretical and practical parts of the subjects among the groupings based on where PC training was obtained (no training, undergraduate training, and graduate or non-university training). The Kruskal–Wallis test result was statistically significant (Kruskal–Wallis chi-square = 13.184, gl = 2, *p*-value = 0.001). Moreover, Dunn’s test for multiple comparisons (using Holm’s method to correct the *p*-value) identified significant differences between specific groups: Degree Vs. No training (adjusted *p*-value = 0.012) and Postgraduate + Non-University Vs. No training (adjusted *p*-value = 0.01). There was, however, no significant contrast in the practical score across groups (Kruskal–Wallis chi-square = 5.111, gl = 2, *p*-value = 0.078).

### 3.2. Training Plan

Out of the 361 documents found in the literature related to basic palliative care training for nurses, five documents were selected. These include the National Consensus Project’s Clinical Practice Guidelines for Quality Palliative Care (NCP Guidelines) 4th edition [41], Competencies And Recommendations for Educating Undergraduate Nursing Students Preparing Nurses to Care for the Seriously Ill and their Families from the End-of-Life Nursing Education Consortium (ELNEC) [42], Foundations in palliative care: A Program of Facilitated Learning for Care Home Staff of the Macmillan Cancer Relief [43], Nursing competencies in palliative care [18] and Recommendations about training in palliative care in nursing degree of the Spanish Palliative Care Nurses Association [17]. The NCP Guidelines establish training in eight domains, and ELNEC adds another three. The complete list of domains include: Structure and processes of care, Physical aspects of care, Psychological and psychiatric aspects, Social aspects of care, Spiritual, religious, and existential aspects of care, Cultural aspects of care, Care of the patient nearing the end of life, Ethical and legal aspects of care, Nursing research, Nursing leadership and Special nursing management courses. The nursing competencies in PC in its training content for the basic level include five blocks: Principles of PC, Communication skills, Symptomatic management and specific care plans, Coping with loss and death, and Ethical and legal aspects. The recommendations on training in palliative care in nursing degree of the Spanish Palliative Care Nurses Association establishes four training modules (PC principles, Communication skills, Symptomatic management and specific care plans, and Legislation and bioethics at the end of life), as does the Macmillan program. The latter program does not include the legislation and bioethics module, but does include the bereavement care module.

The training plan was designed based on the basic competencies in PC and the recommendations for undergraduate training established by AECPAL. It was underpinned by the training needs detected and was aimed at practical applicability, on the basis of the competencies of primary care nurses. It was established in five thematic and content areas, based on the instrument used to detect training needs: PC principles, symptomatic management, coping with loss and death, communication skills, and ethical and legal aspects.

A general objective and five specific objectives coinciding with the training areas were established.

The active teaching methodology turns the student into the protagonist of his or her learning. With a face-to-face approach, problem-based learning was chosen, in which practical cases are presented and solved, and reflective learning and collaborative group work take place. This type of approach achieves greater motivation and participation and it allows students to contrast their points of view and expose their reasoning in each situation. Simulation encourages critical thinking and problem solving, as well as the evaluation of care plans developed in situations that may arise in the professional development of nurses [44].

This type of methodology is considered a tool that has a bearing on change in practice in the field of business, education and health care [45].

It was considered that it would be convenient to offer the training in the area of coping with loss and death to all the nurses in the department, excluding those with advanced training (even though only a third of them passed the score established in the practical part). This training should be carried out during working hours, in groups of 25 people and in five sessions of 3 h and one session of 1 h, on a weekly interval. The total duration of the training program was set at 42 weeks (6 weeks per group).

The teaching profile was established with minimum standards: home-based clinical teachers with advanced training in PC, or experts in communication and bereavement.

The assessment of the effectiveness of the plan was established by means of pre- and post-test evaluation with the INCUE instrument, carried out at the end of the training of all the nurses. It was established that the evaluation of the training plan in the department should be carried out 6 months after the end of the training, in order to measure its practical application. 

The training plan is included in Table S3.

### 3.3. Bereavement Protocol

Three protocols on bereavement care from other care areas were accessed and reviewed. Nine of the 23 documents found in the literature search on protocols and nursing bereavement care were also reviewed. In addition, other documents related to bereavement care from the gray literature were reviewed, including three on bereavement care and follow-up guides and one graduate thesis. 

The protocols reviewed were either not specific to nurses or included interventions based on follow-up telephone contacts [46,47,48].

Some of them establish the defining characteristics of normal and complicated bereavement, as well as risk factors for complicated bereavement [24,25,26] and levels of care [46].

In the documents reviewed, no indications were specified regarding the follow-up time of the bereaved. Gil-Juliá et al. agree that complicated grief usually appears at least 6 months after the death of the significant other [48] and the Inventory of Complicated Grief (ICG) [23], as a screening tool for adaptation to loss, considers this time as the moment from which awareness of what has happened begins. Only one recommendation for bereavement follow-up, 12 months after the funeral, was found in an older document, [49] in which there was no indication of the number of bereavement follow-up visits to be made, nor the time interval between visits. This document did not describe the assessment and intervention to be performed by the nurse at each visit. 

General nursing interventions and interventions in special situations were identified: emotional support, facilitating bereavement, facilitating the expression of grief, spiritual and decision-making support, symptom control, communication and information to the family, and recommendations [50,51] based on different assessments of the functionality and adaptation of the bereaved, using their own assessment forms [52] or self-assessment scales of the home nurse [53].

The support of the bereaved evaluated in another document indicates the need for assessment tools and referral pathways [54].

Nursing assessment and intervention, considered relevant elements by the expert group, were established by consensus and included in the protocol.

The protocol is presented in Table S4.

## 4. Discussion

The results on training needs show greater needs in PHC nurses of the Valencia-Dr. Peset Health Department, compared to a similar study at the national level, one among other respondents with similar sociodemographic characteristics [10,55] and other studies in other areas [31,55]. The nurses of the Department are, in comparison to the national study, less prepared to care for palliative patients (23.53% vs. 40.5%), despite the fact that a higher percentage of them claimed to have basic training (55.89% vs. 45.4%) and the positive influence of training. More training implies more knowledge and more practical application. Since the questions asked were of a basic nature, all educated participants should score similarly. In the current study, the percentage of nurses with no PC training was higher than in the national study (23.53% vs. 13.9%); however, in other Spanish regions, specific PC teams are integrated into PC, possibly providing others with more training and experience. It is reasonable to think that a greater degree of involvement with PC involves greater training.

The results of the INCUE questionnaire of the Department’s nurses also show worse results than those obtained by other Spanish nurses. They showed less knowledge of PC according to the areas of assessment of this resource, although it is greater if we compare them using other resources that measure only areas of knowledge [31] or perception of preparation in the care of a person with palliative needs [11,32]. In addition, it is worth noting an overwhelmingly lower implementation. Spanish nurses passed the questions on practical application in 43.36% of the cases, while departmental nurses only did so in 14.7% of the cases. These data are worrying, as it implies that PC actually reaches very few of the citizens in this area at the first level of care. This justifies the need for a training plan to provide nurses with the necessary training in PC to be effective.

Postgraduate or continuous training seem to provide nurses with greater preparation, based on the results of this study and other studies [10,11,12,28]; the training plan proposed can provide this.

The current trend is towards virtual training, due to its accessibility and flexibility [56]. However, if it is contemplated as the exclusive mode of delivery, it requires revision or it may not reach optimal results in the area of PC competencies [29]. The disadvantages also include the need for a high degree of discipline on the part of the student, the need for high quality teaching materials, scarce feedback and complexity in the evaluation of learning [57].

These data support face-to-face training in small groups with an active teaching methodology, in which the learning strategy focuses on the learner and his or her empowerment through a constructive process. In addition, these training sessions offer the possibility of interaction among the attendees in real time and feedback with teachers and other participants. This will allow the development of implicit, explicit, associative, significant, critical, emotional, observational, experiential, autonomous, discovery, receptive, cooperative and repetitive learning.

Some authors point to the possibility of incorporating incentives into training [58]. However, the increased economic cost of training meant that this option was not considered in the design of the training plan. Instead, training during working hours was established to facilitate and extend the training to as many nurses as possible.

In relation to the content of the plan, the plans found were part of undergraduate training curricula. These, considered as basic training, served as the basis for the curriculum design, and it was decided to structure them in the areas of evaluation of the questionnaire in view of the post-test assessment.

In this study, the practical application of care related to coping with loss and death, including the care and follow-up of the bereaved, was very low, suggesting that nurses in the department do not perform this care. The consequences of not assessing and detecting the early risk factors for complicated bereavement could pose a potential health problem for the bereaved. In the absence of a bereavement care protocol in the department, it was necessary to develop one. Indeed, the consequences of neglect in the care of the bereaved and the early detection of risk factors for complicated bereavement make the existence and implementation of such a protocol and its training essential. This training can be disseminated among PHC nurses, since a good number of them stated that they were unaware of the palliative care protocols of their center.

The present study has limitations in terms of generalization of the results, given that its design was conceived for a specific context and based on the needs of a specific group of nurses. However, the design is reproducible and applicable to other areas, subject to a study of the training needs of the target group. Furthermore, in the assessment of the training needs of the nurses, representativeness of the study population was guaranteed by the recruitment strategy through the coordinators of the centers, which avoided self-selection biases among the participants. Some reviewers consider it a bias not to consider tracking the IP address to ensure a sole response from each participant. However, in the work context, the same IP address is used by multiple subjects; therefore, tracking it and limiting it to a single response does not eliminate this bias and makes it difficult to include participants.

## 5. Conclusions

There is a clear need for PC training and theoretical knowledge among PHC nurses of the Valencia-Dr. Peset Health Department, specifically addressing the practical application of this knowledge. Specific training plans, such as the one proposed, should be designed to improve the applicability of knowledge so that this type of care reaches the population. This training proposal should be implemented by those responsible for the Health Department and subsequently evaluated to assess its effectiveness.

The absence of a bereavement care protocol and the lack of follow-up of the bereaved highlights the need for a protocol in the Department with specific training to address this area and its implementation.

Improvement in the care of people with palliative needs in the first level of health care requires adequate training of the nurses who care for them, so that their knowledge can be the basis for interventions. PHC nurses should be aware of the importance of their care for people at the end of life and the bereaved.

## Data Availability

The datasets used in the current study are available from the corresponding author upon reasonable request.

## Supplementary Materials

The following supporting information can be downloaded at: https://www.mdpi.com/article/10.3390/nursrep13020078/s1, Table S1: Sociodemographic data of the sample; Table S2: Proportion of subjects exceeding the minimum score in knowledge and practical application, according to level of training; Table S3: Training plan; Table S4: Protocol for care of the bereaved; Figure S1: Relationship between the scores of the respondents in the theoretical and practical part of the questionnaire according to their level of training; Figure S2: Score of the theoretical part of the questionnaire in those subjects with basic training, according to time of training, Figure S3: Score of the practical part of the questionnaire in those subjects with basic training, according to time of training.

## Public Involvement Statement

No public involvement in any aspect of this research.

## Guidelines and Standards Statement

This manuscript was drafted adopted a cross-sectional observational design and complies with the STROBE checklist of items for cross-sectional research [59].
