# Supplementary material for: Training Proposal in Palliative Care for Primary Care Nurses in a Health Area in Spain
Source: Nurs Rep. 2023 Jun 11;13(2):890–901. doi: 10.3390/nursrep13020078 (PMC10301754; doi:10.3390/nursrep13020078)
Supplement: Supplementary file 1 [file nursrep-13-00078-s001.zip › nursrep-2335143-supplementary.pdf]

Tables and figures

Table S1. Sociodemographic data of the sample

Sample			
**Variables**		No.=102	
		Mean ± SD	*n (%)*
**Age (years)**		48 ±12	
**Sex**	Women		92 (90.19)
	Man		10 (9.81)
**Maximum level of Professional qualification**	Graduate		72 (70.59)
	Master		9 (8.82)
	Specialist Nurse		19 (18.63)
	Doctor		2 (1.96)
**Position you currently hold**	Center coordinator		11 (10.79)
	Nurse		80 (78.43)
	Pediatric/adult nurse		8 (7.84)
	Community Case Manager		2 (1.96)
	Model nurse in PC		1 (0.98)
**Professional experience in years**		21 ±12	
**PC training**	Yes		75 (73.53)
	No		27 (26.47)
**Level of education in PC**	Advanced (Master or Doctorate)		3 (2.94)
	Intermediate (80-150 hours)		18 (17.65)
	Basic (25-80 hours)		54 (52.94)
	No training		27 (26.47)
**Moment of formation in PC**	Postgraduate university education (Master)		21 (20.58)
	University degree training		23 (22.55)
	Non-university training		31 (30.40)
	No training		27 (26.47)
**How prepared do you feel to work with palliative patients**	Very prepared		2 (1.96)
	Quite prepared		22 (21.57)
	Somewhat prepared		48 (47.06)
	A little prepared		26 (25.49)
	Not prepared		4 (3.92)
**Need for greater PC training**	A lot		19 (18.63)
	Quite		58 (56.86)
	Some		23 (22.55)
	Little		1 (0.98)
	None		1 (0.98)
**Knowledge of PC care protocols in your workplace**	Yes		23 (22.55)
	No		58 (56.86)
	DK/NA		21 (20.59)

Abbreviations: Standard deviation (SD), palliative care (PC), hours (h.), Don't know/No answer (DK/NA).

Table S2. Proportion of subjects who exceed the minimum score in knowledge and practical application, according to the level of training.

INCUE Questionnaire
Minimum score in the Knowledge Block (Theoretical): 18 points (24 total)
Minimum score in the practical application or Practical Block: 90 points (120 total)

**Level of education**	**Advanced** n=3 (2.94%)		**Intermediate** n=18 (17.65%)		**Essential** n=54 (52.94%)		**No training** n=27 (26.47%)	
	**Blocks**							
	**Theoretical (%)**	**Practical (%)**	**Theoretical (%)**	**Practical (%)**	**Theoretical (%)**	**Practical (%)**	**Theoretical (%)**	**Practical (%)**
**Areas**								
PC principles	100	100	100	38.9	100	17.5	83.3	12.5
Symptomatic manegement	100	66.7	77.8	44.4	66.7	21.1	37.5	20.8
Coping with loss and death	100	33.3	94.4	33.3	77.2	5.26	58.3	8.33
Communication skills	100	66.7	88.9	72.2	86.0	31.6	75.0	20.8
Ethical and legal aspects	100	100	94.4	66.7	89.5	28.1	83.3	12.5

Abbreviations: Palliative Care (PC).

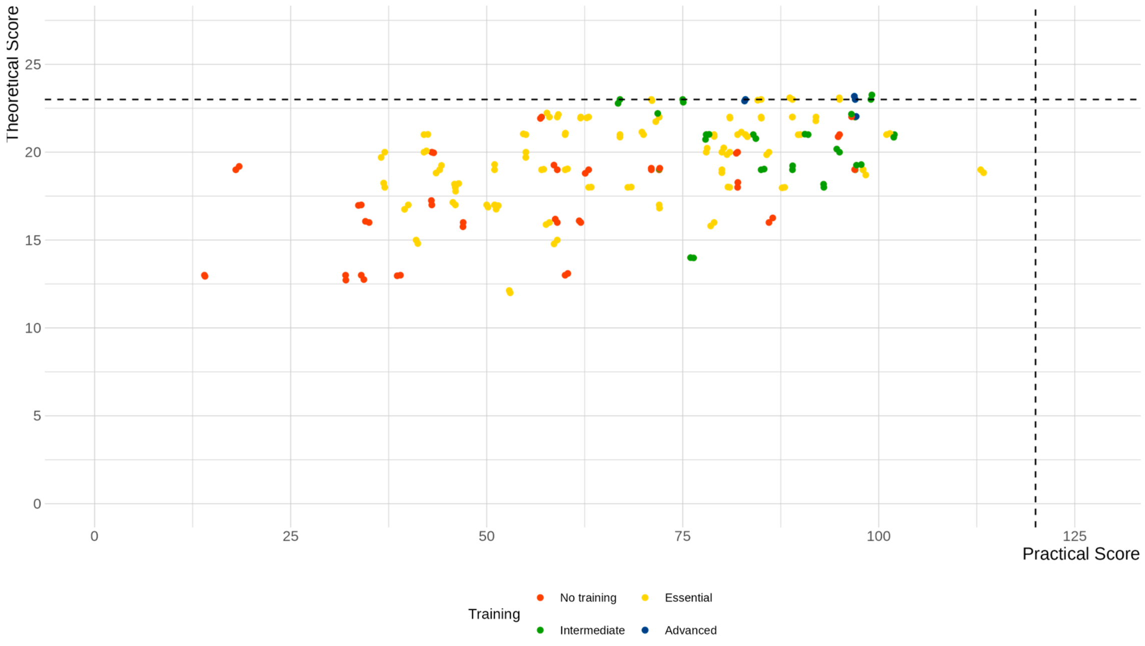

Figure S1: Relationship between the scores of the respondents in the theoretical and practical part of the questionnaire, according to their level of training.

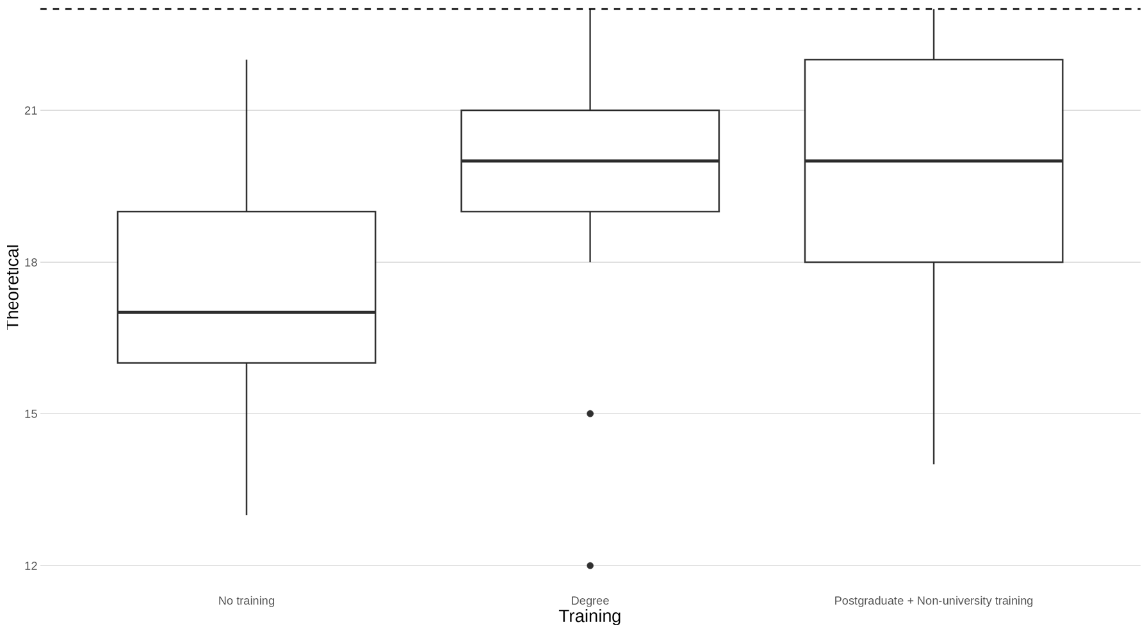

Figure S2: Score of the theoretical part of the questionnaire in those subjects with basic training, according to the moment of training.

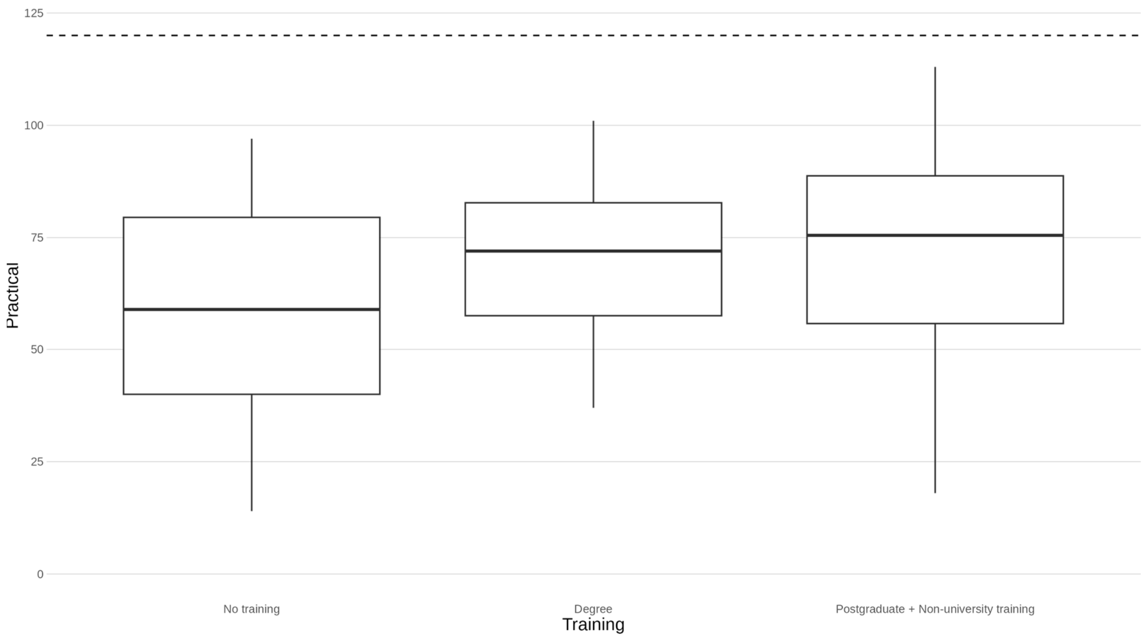

Figure S3: Score of the practical part of the questionnaire in those subjects with basic training, according to the moment of training.

Table S3. Palliative care training plan for primary care nurses in the Dr. Peset-Valencia Health Department.

Phases			Competencies to develop	Contents	Teaching Load
**1.Professional profile to whom the plan is addressed.**	This training plan is aimed at nursing professionals, both novice and expert, in the field of primary care who care for adults in a palliative situation in the Dr. Peset-Valencia Health Department. All nurses, by the fact of being active, have a degree in Nursing or its equivalent.				
**2.Establish the objectives of the plan.**	**General objective:** Acquire knowledge, skills and attitudes necessary for the care of people in advanced illness and/or end of life situation and their families, to increase their quality of life and a better coping with the process of illness and end of life.				
	**Specific objectives:**	*Area 1. Palliative Care Principles*	A1.1 Identify at an early stage people with palliative needs.<br>A1.2 Continuously assess the basic needs of people with palliative needs.<br>A1.3 Understand the concept of the frail person.<br>A1.4 Distinguish between situations of complexity and non-complexity in palliative care.<br>A1.5 Know the existing resources for the referral of highly complex patients	Identification of persons with palliative needs (NECPAL).<br>Comprehensive care. Patient and family needs.<br>Attention to frailty.<br>Diagnosis of complexity in people in palliative situation (IDC-PAL.).	1 session<br>3 hours
**3.Design the agenda and content of the training plan.**		*Area 2. Symptomatic management*	A2.1 Know the management of the subcutaneous route.<br>A2.2 Recognize the last days situation.<br>A2.3 Use symptomatic assessment scales. Assess quality of life.<br>A2.4 Understand the use of non-pharmacological measures.<br>A2.5 Identify refractory symptoms and understand the management of sedation and delirium.<br>A2.6 Assess the symptoms presented by people in advanced and/or end of life, as well as intervene in the main processes.	Routes of administration. Management of the SC route.<br>Last days situation.<br>Assessment scales.<br>Quality of life.<br>Assessment of the efficacy of interventions.<br>Non-pharmacological measures.<br>Sedation: Protocol and action with non-pharmacological measures.<br>Delirium: Protocol and action with non-pharmacological measures.	1 session<br>3 hours
		Area 3. Communication skills	A3.1 Know and incorporate the basics of communication in the nurse-patient relationship. Detect and assess the psychological impact of illness, death and bereavement.<br>A3.2 Know and identify the needs of the family and family claudication.<br>A3.3 Criteria for referral to psychology or social work.	Difficult communication: How to act. What to say and what not to say.<br>Family Claudication. Family needs.<br>Detection of the need for referral to other professionals (social work or psychology).	1 session<br>3 hours
		Area 4. Coping with loss and death	A4.1 Follow up with the bereaved. Know the interval of visits and interventions in each of them.<br>A4.2 Identify situations of risk of complicated bereavement.<br>A4.3 Know the system of referral to mental health.	Bereavement protocol.	1 session<br>3 hours
		Area 5. Ethical and legal aspects	A5.1 Know the bioethical principles and their implication in end-of-life decisions.<br>A5.2 Know how to carry out the process of advance directives and shared care planning.	Preliminary instructions.	1 session<br>1 hours
				Ethical concepts.<br>Decision making.<br>Shared care planning.	1 session<br>3 hours
**4.Decide on the educational strategy and teaching methodology.**	The training will be developed through an active methodology, with the presentation and resolution of clinical cases in the course of the session, as well as the simulation of healthcare activity. Attendees will be supported by theoretical content to help them solve the problems posed and its discussion, as well as help from the teacher.<br>The 173 PA nurses of the Department will be distributed in 7 groups of a maximum of 25 people per group.				
**5.Define the profile of the teachers and their selection according to resources.**	The teachers will be nurses with expertise of more than 5 years in palliative care and with clinical experience in home care.<br>In the area of communication skills and coping with loss and death, the teachers will be clinical psychologists with expertise in communication and bereavement.				
**6.Establish the outcome evaluation systems.**	The evaluation of the results will be carried out by means of the INCUE instrument. The measurement with this instrument will be carried out 6 months after the end of training.<br>The general criteria for evaluating the results of the training was established as the absence of statistically significant difference between passing the theoretical part of the instrument and the practical part. There will be an evaluation of the teaching by the attending students.				
**7.Schedule of the training activity.**	The training will have a duration of 6 weeks per group. It will be developed in 6 training sessions, of which 5 will be of 3 hours duration and 1 of 1 hour duration.<br>The total time period necessary to carry out the training of the PA nurses of the Department is estimated at 42 weeks.				
**8.Prepare a report on the estimated cost of the plan.**	The cost of the plan will be made up in part by the expenditure on human resources involved in releasing the nurses in training from their tasks during the training period.<br>Another part of the cost will be made up by the assistance of the teachers. This will include transportation to attend the sessions, their fees and their lodging if they need to stay overnight in order to carry out the training. The teachers will be paid 20 euros per hour if it is outside their working hours. In total there are 16 hours for 7 groups, which amounts to 2240 euros. Material and training facility of the center itself.				

Table S4. Protocol for support, early identification and referral of people at risk of complicated beravement of Dr. Peset-Valencia Heath Department.

Stage	Execution time	Type of intervention	Objective	Tools	Intervention	Levels of intervention	Professional who performs it
Stage 1<br>First bereavement visit	One month after death	Home visit	Accompaniment and assessment of risk factors for complicated bereavement.<br>Assessment of personal and support resources.<br>Assessment of level of functionality.<br>Assessment of the degree of adaptation to loss.	Interview and assessment of risk factors**:<br>❖ Relational factors:<br>➢ Loss of child, partner, parent at early age and/or sibling in adolescence<br>➢ Dependent relationship of the survivor to the deceased<br>➢ Difficult adaptation to the change of role<br>➢ Conflicted or ambivalent relationship. Unexpressed mixed feelings of love/hate<br>❖ Circumstantial factors:<br>➢ Youth of the deceased<br>➢ Sudden loss, accident, homicide, suicide, uncertain and/or multiple<br>➢ Duration of illness and agony<br>➢ Non-recovery of the corpse. Body with damaged or deformed appearance<br>➢ Inability to see the body Painful memory of the process: inadequate relationships with health personnel, diagnostic difficulties, poor symptom control or others.<br>➢ Stigmatized death (AIDS, socially unaccepted partner)<br>❖ Personal factors:<br>➢ Survivor's old age or youth<br>➢ Lack of stress management resources<br>➢ Previous physical and/or mental health problems (anxiety, depression, suicide attempts, alcoholism, and personality disorder)<br>➢ Lack of hobbies and interests<br>➢ Previous unresolved duels<br>➢ Very intense reactions of anger, bitterness and guilt<br>➢ Subjective assessment of lack of resources to cope with the situation<br>❖ Social factors:<br>➢ Absence of social/family support system or relationship conflicts with them<br>➢ Scarce socio-economic resources<br>➢ Responsibility for young children<br>➢ Other stressors: labor conflict, type of interrupted life project, etc.<br>Non-verbal language assessment.<br>Assessment of physical appearance of the bereaved.<br>Assessment of the physical space: cleanliness, lighting and care.	1. What has been the nature and impact of the loss?<br>Identification of risk factors for complicated bereavement.<br>Complicated risk factor assessment.<br>2. How is the person experiencing the grieving process?<br>Functionality assessment:<br>a. Adjusted emotional manifestation: presence of adequate expressions of mourning in duration and intensity, according to the reference culture (see table 1).<br>b. General functioning level: has recovered and/or maintains social relationships, work, leisure, self-care behaviors (walks, eating...).<br>3. Questions to ask on the first visit.<br>- How are you experiencing the grieving process? Do you understand your emotions as normal in this process?<br>- Have you been calm in the last few days?<br>- What is the most difficult thing for you at this stage?<br>- What things/people help you right now?<br>- Have you recovered your work/leisure activities? What entertains you?<br>- What self-care measures do you take? Exercise, rest, food, reading, relaxation, etc.<br>- How do you rest at night? How did you rest before the loss?<br>- Do you take something to help you rest?<br>- Tell me what you ate yesterday.<br>- What is the most important thing for you right now?	Level I: Basic support<br>*Bereaved with symptoms adjusted to an adaptive process and without risk factors for complicated bereavement.<br>Basic advice on the emotional process and grief hygiene.	Level I:<br>Nurse<br>Doctor
Stage 2<br>Second bereavement visit	Three months after death	Home visit	Accompaniment and identification of complications in grief.<br>Assessment of personal and support resources.<br>Assessment of level of functionality.<br>Assessment of the degree of adaptation to loss.<br>Identification of the areas of emotional difficulty of the bereaved.	ICG Questionnaire***	In the emotional manifestation: perception of the emotional experience of the bereaved person.<br>-Do you feel that you can adequately handle your emotions? Do you feel that you are overflowing on an emotional level? Do you feel that you need help to understand your grieving process?<br>In general functioning: he/she has recovered and/or maintains social relationships, work, leisure, self-care behaviors (walks, eating...).	Level I and/or II<br>Level I: Basic support*<br>Level II: Specific support<br>Patient without complicated risk factors and with difficult-to-manage emotional symptoms (ICG>25)<br>Referral to a professional grief specialist or grief support group associations.	Level I:<br>Nurse<br>Doctor<br><br>Level II:<br>Professional grief specialist<br>Bereavement associations
Stage 3<br>Third bereavement visit	Six months after death	Home visit	Accompaniment, identification of complications in grief and referral to a specific resource.	ICG Questionnaire***	Identify areas of emotional difficulty for the bereaved person.<br>➢Before a normalized mourning process (ICG<25), Level I intervention.<br>➢If ICG>25, refer to Mental Health, Level III of intervention.	Level I and/or III<br>Level I: Basic support*<br>Level III: Clinical intervention<br>Bereaved with risk factors for complicated bereavement and difficult-to-manage emotional symptoms (ICG>25).	Level I:<br>Nurse<br>Doctor<br><br>Level III:<br>Professional grief specialist<br>Mental health
Stage 4<br>Fourth bereavement visit	One year after death	Home visit	Accompaniment, identification of complications in grief and referral to a specific resource.	ICG Questionnaire***	Identify areas of emotional difficulty for the bereaved person.<br>➢Before the normalized mourning process (ICG <25), closure of the care process (Discharge).<br>➢If ICG>25, refer to Mental Health, Level III of intervention.	Level I and/or III<br>Level I: Basic support*<br>Level III: Clinical intervention<br>Bereaved with risk factors for complicated bereavement and difficult-to-manage emotional symptoms (ICG>25).	Level I:<br>Nurse<br>Doctor<br><br>Level III:<br>Professional grief specialist<br>Mental health

**SECPAL Guide for bereaved family members [Internet]. 2006 [accessed November 28, 2021]. Available at: https://www.bdv.cat/sites/default/files/common/Salut/guia_per_a_familiars_en_dol.pd.

***Inventory of Complicated Grief (ICG): *Complicated grief: total score >25. Taken from Limonero García JT., Lacasta Reverte M., García García JA., Maté Méndez J., Prigerson H.g. Spanish adaptation of the inventory of complicated grief, Palliative Medicine, 2009, vol 16, no 5, pages 291-29*
